# Research Hotspot and Trend of Employee Creativity Based on Bibliometric Analysis

**DOI:** 10.3389/fpsyg.2022.914401

**Published:** 2022-10-06

**Authors:** Yang Shi, Hong-yuan Zhang

**Affiliations:** ^1^School of Business, Changshu Institute of Technology, Suzhou, China; ^2^School of Business, Jiangsu Ocean University, Lianyungang, China

**Keywords:** employee creativity, knowledge graph co-citation relationship, co-word clustering, bibliometric analysis, human resource management

## Abstract

Employee creativity is the foundation of organizational competitiveness, and it is also the important theme of creativity research. Based on the knowledge graph theory, this article uses the Citespace software to conduct a bibliometric analysis of 1,168 importance literature from the Web of Science and draws the co-citation and co-word clustering knowledge graph to explore research hotspots and trends of employee creativity. The research found that: First, the research on the connotation, dimensions, and behavior of employee creativity is still in the initial stage; second, the research on the psychological, behavioral, and management factors that affect employee creativity is the key content of common concern; and third, it needs to comprehensively consider relevant factors from the combination of individuals, groups, and organizations about employee creativity research. On this basis, this article proposes the main directions for future research.

## Introduction

Employee creativity is not only the foundation of organizational competitiveness but also the important theme of creativity research. In recent years, scholars have conducted many studies on what is employee creativity and how to improve employee creativity. From the definition (Hirst et al., 2009) and model expansion to variable verification (Keem et al., 2018), many high-quality studies have been published (Shalley and Gilson, 2004; Zhou and Hoever, 2014; Lin et al., 2016). However, employee creativity is in the initial stage of research, and the concept connotation and measurement standard of employee creativity are not unified, which limits further research on employee creativity. Therefore, it is necessary to distinguish the research status and identify the research frontier and development trend of employee creativity.

Based on the perspective of literature review, there are two ideas. First, the review analyzes the theoretical basis, research theme, and knowledge structure from the perspective of qualitative analysis (Anderson et al., 2014). Second, it analyzes the number of documents, authors and institutions, keyword distribution, and citation from the perspective of quantitative analysis. At present, there are few relevant reviews of dynamic analysis in the literature, and it is difficult to reflect the overall development of creativity research in recent years. Meanwhile, literature review lacks further combining of important literature, especially the recent literature, which is difficult to reflect the latest progress in employee creativity research.

Using the relevant methods of bibliometrics, this article systematically studies the keywords of creativity research and analyzes the hotspot drift of high-frequency keywords, which will provide reference for academic research in related fields. With CiteSpace, the scientific visualization software, this article systematically analyzes the literature that is published in international journals, verifies the previous research conclusions, and shows the development and evolution of employee creativity research. On this basis, it predicts the future research trend and provides reference for academic research.

## Materials and Methods

### Search Strategy and Refined Data

The data source takes the target journals in the Web of Science database; those are 50 authoritative economic and management journals (FT50) identified by *Financial Times*, which are used in compiling FT research rankings, including global MBA, EMBA, and online MBA rankings, and 24 mainstream top journals (Utd24) used by the Naveen Jindal school of management of UT Dallas in ranking the top 100 business schools in the world. Some of them overlap, total journals are 64. In addition, considering the specialty of creativity research, journals, such as *creativity research journal* and *Journal of creative behavior*, are included in the database.

Sort out the literature published with “employee creativity” in title, abstract, and keywords, and the time window is up to December 1, 2021. After confirming the source of research samples and deleting irrelevant items manually, 1,168 studies are finally obtained. The keywords of the literature are screened and replaced as the main basis for further research.

The top 10 research results on employee creativity published in the international top journal are as follows: *Creativity Research Journal* (234), *Journal of Creative Behavior* (208), *Journal of Business Research* (64), *Academy of Management Journal* (59), *Journal of Organizational Behavior* (54), *Journal of Applied Psychology* (53), *Harvard Business Review* (51), *Leadership Quarterly* (41), *Journal of Business Ethics* (40), and *Journal of Management* (35). From the perspective of journal distribution, it is mainly concentrated in relevant journals, such as management, psychology, creativity, behavior, and ethics, reflecting that the research on employee creativity has been widely recognized and valued by the academic community.

### Research Methods

Bibliometric analysis can quantitatively describe the characteristics and scholarly impact of citation classics (Castillo-Vergara et al., 2018; Donthu et al., 2021). Understanding the characteristics of highly cited literature may help scholars who wish to submit and publish effectively. It also can reveal the research theme and the current situation and describe the basic research literature, research frontier, and research development, which is very helpful for scholars in a specific field.

**FIGURE 1:**
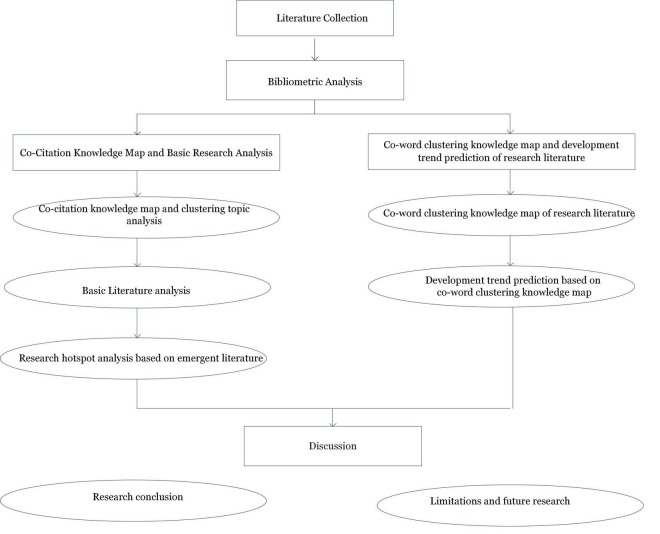
Research design.

CiteSpace is a kind of miscellaneous shareware software developed by Chaomei Chen in Drexel University. It is a very practical visual analysis in scientific citation analysis, which can identify and display new trends and trends of scientific development. This article used the CiteSpace software to conduct a bibliometric analysis on the literature related to employee creativity. Through the quantitative analysis of relevant literature, which draws the co-citation knowledge map and co-word clustering knowledge map, it identifies the current situation, hotspots, and trends of employee creativity research and provides direction for the development of relevant research in the future. Research design is shown in Figure 1.

## Results

### Co-citation Knowledge Map and Basic Research Analysis

(1) Co-citation knowledge map and clustering topic analysis

**FIGURE 2:**
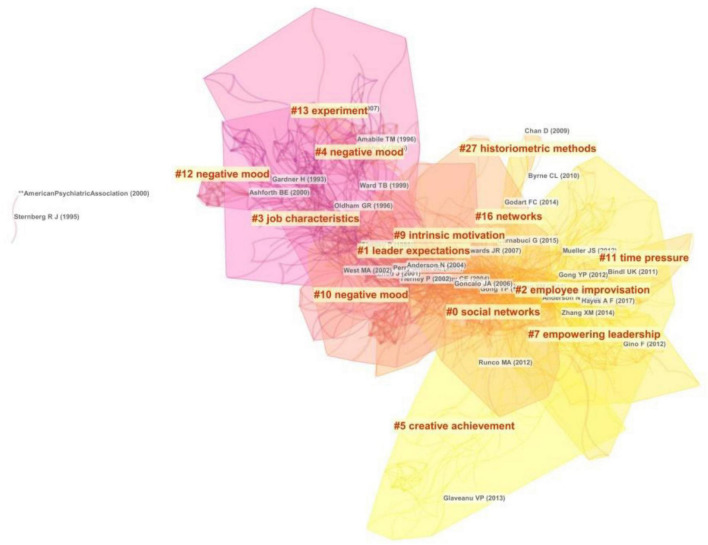
Co-citation knowledge map in 2000–2021.

The cited documents of 1,168 articles in international journals were sliced annually, and the top 50 cited literature were extracted to generate a knowledge map network. After the network clustering is stable, a total of 14 large modules are obtained, including 947 network nodes, 3,048 connection edges, network density of 0.0068, module degree of 0.6567, and homogeneity coefficient of 0.3532. The information and research contents of 14 large clustering modules are shown in Figure 2.

From Figure 2, it can be seen that the homogeneity coefficient of each cluster is large, indicating that the similarity of research topics is high. Further research found that the research contents of related clustering literature have certain similarities and can be merged. Clustering 0 and 16 mainly study the impact of social networks on employee creativity from the perspective of group behavior, and it can be summarized as group factors affecting employee creativity (#1); clustering 2 and 7 mainly study the impact of leadership traits and behaviors on employee creativity from the perspective of team leadership, such as empowering leadership and transformational leadership. It can be summarized as organizational factors affecting employee creativity (#2); clustering 3 mainly studies the impact of work characteristics on employee creativity from the perspective of work environment. It can be summarized as environmental factors affecting employee creativity (#3); clustering 4, 5, 9, 10, 11, and 12 mainly study the impact of individual non-intellectual factors on employee creativity from the perspective of creative achievement, internal motivation, negative mood, time pressure, etc. It can be summarized as individual factors affecting employee creativity (#4); clustering 13 and 27 mainly study the research methods and paths of employee creativity from the perspective of empirical analysis. It can be summarized as research methods of employee creativity (#5). From the time and research dimension of literature clustering, the research on employee creativity has entered the peak after 2000, and the research on individual factors has always been the hotspots. Research contents based on co-citation literature clustering are shown in Table 1.

**TABLE 1:** Research contents based on co-citation literature clustering.

Clustering	Scale	Homogeneity coefficient	Time	Content	Research dimension
0	149	0.558	2009	social networks	#1
1	114	0.721	2004	leader expectations	#2
2	100	0.706	2013	employee improvisation	#4
3	94	0.824	1997	job characteristics	#3
4	55	0.886	1998	negative mood	#4
5	54	0.922	2015	creative achievement	#4
7	40	0.883	2013	empowering leadership	#2
9	23	0.85	2007	intrinsic motivation	#4
10	17	0.941	2000	negative mood	#4
11	17	0.974	2010	time pressure	#4
12	14	0.988	2000	negative mood	#4
13	6	0.991	2006	experiment	#5
16	5	0.981	2011	networks	#1
27	3	0.996	2009	historiometric methods	#5

*#1 Group factors; #2 organizational factors; #3 environmental factors; #4 individual factor; and #5 research methods.*

(2) Basic literature analysis

Basic literature is widely accepted and cited by academia, and it is an important symbol to reflect the research basis in this research field. The frequency and centrality of the cited literature are higher; the guiding significance is stronger for the follow-up research. Through the analysis of the co-citation knowledge map of the literature on employee creativity, the relevant basic literature is obtained, as shown in Table 2.

**TABLE 2:** Basic literature based on co-citation (Citation Frequency top 10).

Frequency	Centrality	Particular year	Author	Subject	Research dimension
94	0.05	2014	Anderson N	Innovation and Creativity in Organizations: A State-of-the-Science Review, Prospective Commentary, and Guiding Framework	#5
71	0.05	2009	Gong YP	Employee Learning Orientation, Transformational Leadership, and Employee Creativity: The Mediating Role of Employee Creative Self-Efficacy	#2,#4
69	0.05	2010	Zhang XM	Empowering Leadership And Employee Creativity: The Influence of Psychological Empowerment, Intrinsic Motivation, and Creative Process Engagement	#2,#4
59	0.03	2004	Shalley CE	What leaders need to know: A review of social and contextual factors that can foster or hinder creativity	#5
54	0.05	2014	Zhou J	Research on workplace creativity: a review and redirection	#5
54	0.04	2011	Grant AM	The necessity of others is the mother of invention: Intrinsic and prosocial motivations, perspective taking, and creativity	#4
47	0.05	2007	George JM	Dual Tuning in a Supportive Context: Joint Contributions of Positive Mood, Negative Mood, and Supervisory Behaviors to Employee Creativity	#2,#4
43	0.1	2009	Shalley CE	Interactive effects of growth need strength, work context, and job complexity on self-reported creative performance	#3
42	0.06	2006	Perry-Smith JE	Social yet creative: The role of social relationships in facilitating individual creativity	#1
41	0.04	2009	Hirst G	A cross-level perspective on employee creativity: Goal orientation, team learning behavior, and individual creativity	#1, #2

*#1 Group factors; #2 organizational factors; #3 environmental factors; #4 individual factor; and #5 research methods.*

As shown in Table 2, the basic research literature is mainly concentrated in 2006–2014, which reflects an important foundation for the study of employee creativity and promotes the empirical verification of relevant studies. From the content of highly cited literature, it is mainly divided into the following three aspects: first, it is the analysis and prospect of the concept, connotation, and dimension of employee creativity. This kind of literature often adopts the form of qualitative review, which has strong methodological guidance for follow-up research. For example, Anderson et al. (2014) combed several pioneering theories of creativity and innovation, summarized the research ideas of multidimensional and multilevel integration, pointed out the research path of “individual-team organization” integration, and put forward 11 main themes and 60 specific problems for future research. Zhou and Hoever (2014) reviewed the empirical results of workplace creativity research in the field of organizational psychology and management since 2000. They discussed the impact of the interaction between participants and their situation on employee creativity and put forward the future research direction and practical significance. Second, it involves the research on individual, group, organization, and environmental factors affecting employee creativity. For example, Gong et al. (2009) studied the impact of employee learning orientation and transformational leadership on employee creativity and took creative self-efficacy as an intermediary variable. Zhang and Bartol (2010) analyzed the relationship between authorized leadership and employee creativity and analyzed the impact of psychological authorization, intrinsic motivation, and creative process participation on employee creativity. Through the investigation of employees and supervisors of one large information technology company in China, it is found that authorized leadership has a positive impact on psychological empowerment and affects employee internal motivation and creative process participation. Third, it is the research on the formation mechanism of employee creativity, which involves the comprehensive use of multidimensional and multilevel variables. For example, George and Zhou (2007) developed a dual regulatory view to explore how positive and negative emotions interact to affect creativity in a supportive environment. The research shows that when the supervisor provides a supportive environment for employees and the positive emotion is high, there is a strong positive relationship between negative emotion and creativity. Hirst et al. (2009) studied the relationship between goal orientation, team learning behavior, and employee creativity based on cross-level analysis. The research shows that there is a non-linear interaction between individual learning orientation and team learning behavior. In the team with high level of team learning behavior, there is a positive relationship between learning orientation and employee creativity.

### Research Hotspot Analysis Based on Emergent Literature

The emergent literature has high research value. The emerging time is the symbol of the recognition, and the research field can reflect the current research hotspot. According to the co-citation knowledge map, the top 10 emergent literatures of employee creativity are obtained, as shown in Table 3.

**TABLE 3:** Emergent literature based on co-citation (top 10).

Author	Particular year	Strength	Start time	End time	Subject	Research dimension
Oldham GR	1996	18.3002	2000	2004	Employee creativity: Personal and contextual factors at work	#3, #4
Tierney P	1999	17.5851	2001	2007	An examination of leadership and employee creativity: The relevance of traits and relationships	#2
Zhou J	2001	15.0388	2003	2009	When job dissatisfaction leads to creativity: Encouraging the expression of voice	#4
Tierney P	2002	15.409	2004	2010	Creative self-efficacy: Its potential antecedents and relationship to creative performance	#4
Shalley CE	2004	23.9414	2006	2012	What leaders need to know: A review of social and contextual factors that can foster or hinder creativity	#3
George JM	2007	15.1541	2010	2015	Dual Tuning in a Supportive Context: Joint Contributions of Positive Mood, Negative Mood, and Supervisory Behaviors to Employee Creativity	#2, #4
Gong YP	2009	16.9154	2011	2017	Transformational Leadership, and Employee Creativity: The Mediating Role of Employee Creative Self-Efficacy	#2, #4
Zhang XM	2010	17.3336	2012	2018	Empowering Leadership And Employee Creativity: The Influence of Psychological Empowerment, Intrinsic Motivation, and Creative Process Engagement	#2, #4
Zhou J	2014	16.3813	2017	2021	Research on workplace creativity: a review and redirection	#5
Anderson N	2014	30.0697	2017	2021	Innovation and Creativity in Organizations: A State-of-the-Science Review, Prospective Commentary, and Guiding Framework	#5

*#1 Group factors; #2 organizational factors; #3 environmental factors; #4 individual factor; and #5 research methods.*

According to Table 3, the emergent literature was first published in 1996, and the emerging time was concentrated after 2000. For example, Oldham and Cummings (1996) studied the relationship between employee personal characteristics, organizational environment characteristics (i.e., work complexity, supportive supervision, and regulatory supervision) and employee creativity. From the perspective of emergent literature strength, intensity values are higher than 15, which reflect these literatures have played an important role in the basic research of employee creativity. For example, Shalley and Gilson (2004) studied the environment factor that may promote or hinder employee creativity at the work, group, and organizational levels, which include leadership, human resources practice, and working in supporting environment. From the perspective of emergent literature time, the emerging period is about 5 years, which also shows these literatures have a high academic value. It is found that the emergence time of Anderson et al. (2014) and Zhou and Hoever (2014) has not ended, indicating that the research on employee creativity is still in a hot research state. From the perspective of emergent literature dimension, the research dimension concentrates on organizational factors (#2), such as leadership and supervisory behaviors; environmental factors (#3), such as contextual factors; and individual factors (#4), such as personal, mood, psychological empowerment, creative self-efficacy, and intrinsic motivation. In addition, the variable design method of “individual group organizational” provides ideas for the research of employee creativity.

### Co-word Clustering Knowledge Map and Development Trend Prediction of Research Literature

**(1) Co-word clustering knowledge map of research literature**

Co-word cluster analysis can get the evolution relationship of research literature and present the evolution process of keywords, which will help scholars identify the research frontier and distinguish the research development trend. Using the CiteSpace software, the co-word clustering knowledge map of employee creativity is obtained, as shown in Figure 2.

**FIGURE 3:**
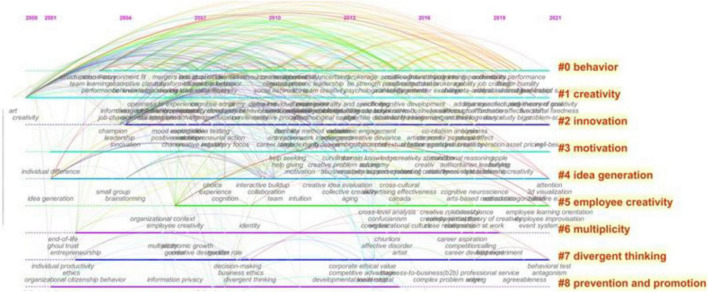
Co-word clustering knowledge map of employee creativity research from 2000 to 2021.

As can be seen from Figure 3, the co-word clustering results of employee creativity divide the keywords of the research literature into 9 categories. The keywords of each cluster are as follows: clustering #1 and #2 are related to the concept of creativity, which include creativity and innovation; clustering #5 is related to the object of creativity; clustering #0, #3, #4, and #7 are related to individual factors that affect employee creativity, which include behavior, motivation, idea generation, and divergent thinking; clustering #6 is related to the group factors that affect employee creativity, which indicates multiplicity; and clustering #8 is related to the mechanism that affects employee creativity.

**(2) Development trend prediction based on co-word clustering knowledge map**

Through co-word cluster analysis, 21 high-frequency keywords related to employee creativity are obtained with centrality higher than 0.03, which are specifically divided into the following six aspects: employee creativity–related concepts, research objects, research methods, and related factors affecting employee creativity (i.e., individual factors, group factors, and organizational factors), as shown in Table 4.

**TABLE 4:** High-frequency keywords based on co-word clustering.

Category	Key word	Frequency	Centrality	Category	Key word	Frequency	Centrality
EC related concepts	creativity	267	0.76	Individual factors	motivation	41	0.12
	innovation	54	0.18		creative self-efficacy	35	0.08
	performance	50	0.17		personality	21	0.04
	new product development	7	0.03	Group factor	behavior	36	0.1
Research object	employee creativity	36	0.1		knowledge sharing	28	0.09
	team creativity	25	0.03		work engagement	21	0.07
					team learning	13	0.05
Research Method	multilevel	18	0.04		leader-member exchange	16	0.03
					diversity	18	0.03
					social network	26	0.05
				Organizational factors	organizational culture	19	0.05
					leadership	32	0.07
					transformational leadership	27	0.04
					supervisor	12	0.03

(1) Concepts related to employee creativity (E-C) include innovation (centrality = 0.18), performance (centrality = 0.17), and new product development (centrality = 0.03). Taking performance as an example, it explores antecedent variables that affect employee creativity, such as job performance, team performance, firm performance, innovative performance, and task performance.

(2) The research objects include employee creativity (centrality = 0.1) and team creativity (centrality = 0.03). From the research level, early research mainly focused on the relevant variables affecting employee creativity from a single level (Zhou and Hoever, 2014). More variables reflecting individual characteristics, group behavior, and organizational factors have been included in the research category (Anderson et al., 2014).

(3) Research methods include multilevel (centrality = 0.04), model, mediation role, mediating role, and meta-analysis. From the research path, the empirical research on antecedent variables, outcome variables, intermediary variables, and constraint variables is more mature. Since the pioneering work of Bowen and Ostroff (2004), multilevel research has been attached in the research of strategic human resource management. In recent years, research based on multilevel and multivariable has become the mainstream (Chang et al., 2014; Gu et al., 2018; Song et al., 2020).

(4) Individual factors affecting employee creativity include motivation (centrality = 0.12), creative self-efficacy (centrality = 0.08), and personality (centrality = 0.04), which play an important role in the field of personal perception and self-consciousness. Taking motivation as an example, some scholars expanded from the perspectives of cognitive motivation, achievement motivation, innovation motivation, task motivation, prosocial motivation, auto motivation, and creative motivation (Grant and Berry, 2011; Liu et al., 2013; Li P. et al., 2020). The recognition and re-understanding of personality also deserve attention. In recent years, some scholars expanded from the perspectives of active/compliance personality, task/fun personality, achievement/ordinary personality, and creative personality (Qian et al., 2010; Jiang and Gu, 2015). In recent years, perception research has gradually become a hotspot, especially the research on individual perception (Kilroy et al., 2016; Escribá-Carda et al., 2017; Tang et al., 2020).

(5) Group factors affecting employee creativity include behavior (centrality = 0.1), knowledge sharing (centrality = 0.09), work engagement (centrality = 0.07), team learning (centrality = 0.05), leader–member exchange (centrality = 0.03), diversity (centrality = 0.03), and social network (centrality = 0.05). From the perspective of group interaction, team characteristic factors (i.e., cognition, gender, and knowledge diversity), team interaction factors (i.e., knowledge sharing, team learning, team commitment, and team conflict), and team support factors (i.e., social network, shared mental model, and interactive memory system) have an impact on employee creativity. Taking behavior research as an example, in recent years, researchers have paid attention to positive organizational behaviors, such as organizational citizenship behavior, prosocial behavior, voice behavior, and feedback behavior (Eldor and Harpaz, 2016; Han et al., 2020), as well as complex organizational behaviors, such as extra role behavior, unethical behavior, and device behavior (Kim et al., 2009; Keem et al., 2018). Taking conflict as an example, some scholars also put forward the concepts of relationship conflict, status conflict, task conflict, cognitive conflict, and work–family conflict. At the same time, on the basis of leader–member exchange, some scholars have added contents, such as leader-member exchange (LMX), leader-leader exchange (LLX), and team-member exchange (TMX).

(6) Organizational factors affecting employee creativity include organizational culture (centrality = 0.05), leadership (centrality = 0.07), transformational leadership (centrality = 0.04), and supervisor (centrality = 0.03). In general, organizational orientation factors (i.e., achievement goal orientation, learning goal orientation, market orientation, entrepreneur orientation, entrepreneurship orientation, performance orientation, and cultural orientation), organizational management factors (i.e., human resource management system, leadership behavior, and organizational motivation), and organizational climate factors (i.e., organizational justice, organizational pressure, organizational culture, and organizational politics) are the key factors affecting employee creativity. Taking leadership as an example, transformational leadership and empowering leadership have always been research hotspots. In recent years, some scholars have also studied from positive perspective (Tse et al., 2018), such as service leadership, authentic leadership, charismatic leadership, shared leadership, supportive leadership, benevolent leadership, and ethical leadership, while others have studied from a negative perspective, such as self-serving leadership and paradoxical leadership (Hou et al., 2019; Peng et al., 2019; Wang et al., 2019). In addition, human resource management systems are the traditional research topics in the field, such as high-performance work system and high-commitment work system (Zhou et al., 2019; Li H. et al., 2020; Li P. et al., 2020).

## Discussion

### Research Conclusion

Bibliometric analysis can understand the characteristics of highly cited studies and help authors to submit and publish effectively. At present, it is the most widely accepted method to assess the merits of the specific field. Using the bibliometric method and the scientific visualization software CiteSpace, this article makes co-citation knowledge map and co-word clustering knowledge map on employee creativity in the core collection of Web of Science. It identifies research topics and basic research literature, cardings research hotspots, and research trends, which can play an important reference for variable selection, factor classification, and cross-level analysis of future research.

First, the basic literature and emergent literature of employee creativity show that the research on the connotation, dimension, and behavior performance of employee creativity is still in its infancy. From the perspective of research theme, employee creativity is closely related to innovation, performance, and new product development. The research literature of Anderson et al. (2014) and Zhou and Hoever (2014) also reflects that the research on employee creativity has great significance to stimulate employees’ innovation potential and improve organizational innovation performance.

Second, the co-word clustering of employee creativity shows that research hotspots include individual, group, and organizational factors that affect employee creativity, and the attention from individual and group behavior processes continues to increase. From the perspective of research variables, it involves the psychological, behavioral, and management factors that affect employee creativity, which is also the key content of common concern.

Third, in the early stage, the research methods of employee creativity mainly focused on single-level and multivariable research. In the recent period, it has shown that multilevel and multivariable research has become the mainstream. Therefore, it is necessary to comprehensively consider the relevant factors that affect employee creativity under the combined action of individuals, groups, and organizations.

### Limitations and Future Research

Although this study adopts a multisource and paired study design, there are still some limitations. First, the data source is 1,168 important studies in the database of Web of Science database, but the scope of the literature is not enough to fully reflect the overall picture of employee creativity. Second, it selects and replaces the keywords in the literature as the main basis. However, some articles may not be included in the research field because of the differences in research topics. Third, although quantitative analysis has been drawn through co-citation and co-word clustering knowledge map, the research needs to strengthen and combine with other quantitative analysis methods. Future research directions include the following:

(1) The research on multisystem innovation that influences employee creativity. At present, facing the impact of new technologies and new platforms, creativity research has undergone new changes in research basis and research content. Therefore, it is necessary to fully understand the new changes and trends in the development human resources management, such as artificial intelligence and sharing economy. Around this research theme, research focuses on technological and organizational change, organizational behavior and leadership, employee incentive, and institutional environment, which further enhances the research dimension and application field about employee creativity. For example, new organizational forms influence on employee creativity, such as borderless or platform organization; the conflict of multiple employment relationships in organizations; and the relationship between talent flow and employee creativity from the perspective of social network.

(2) The research on multivariable combination that influences employee creativity. From the perspective of creative subject, creative behavior, and creative environment, it will consider variables, such as individual motivation, group behavior, organizational atmosphere, and environmental factors, respectively; individual factors (i.e., personality, motivation, interest, emotion, personality, and emotion), group behavior factors (i.e., team structure, team atmosphere, and team interaction), and organizational environment factors (i.e., organizational orientation, organizational pressure, and organizational politics). On this basis, it studies the comprehensive application of positive–negative effects, combination effects, and difference effects, which can analyze the complex characteristics and internal mechanism about employee creativity.

(3) The research on multilevel span that influences employee creativity. At present, the research level of employee creativity is gradually manifested from individual to team, organization, and then to multilevel. In this sense, it is more necessary to establish a cross-layer analysis framework model. Anderson et al. (2014) pointed out that creativity research includes analytical perspectives. Combined with the research path of employee creativity, possible research directions include individual-team (I-T), which reflects individual employee ideas or suggestions are adopted on team creativity, such as employee work pressure and psychological empowerment; team-individual (T-I), which reflects working group affecting on individual creativity, such as team transformational leadership and organizational learning; team-organization (T-O), which reflects team process affecting on organizational creativity, such as leader–member exchange and entrepreneur orientation; and organizational-team (O-T) reflects organizational processes affecting on team creativity, such as organizational innovation atmosphere and performance incentive. Combined with the research perspective of multilevel analysis, it can more comprehensively reflect the mechanism of employee creativity generation and stimulation.

## Author Contributions

YS put forward the conceptual model, wrote the manuscript, and collected the data. H-YZ made valuable suggestions for both the initial draft and subsequent revisions. Both authors contributed to the article and approved the submitted version.

## Conflict of Interest

The authors declare that the research was conducted in the absence of any commercial or financial relationships that could be construed as a potential conflict of interest.

## Publisher’s Note

All claims expressed in this article are solely those of the authors and do not necessarily represent those of their affiliated organizations, or those of the publisher, the editors and the reviewers. Any product that may be evaluated in this article, or claim that may be made by its manufacturer, is not guaranteed or endorsed by the publisher.
